# A Case of Dry Gangrene

**Published:** 1854-09

**Authors:** B. A. Gailbreath

**Affiliations:** Louisville, Ky.


### ART. III.—A CASE OF DRY GANGRENE.

BY DR. B. A. GAILBREATH, OF LOUISVILLE, KY.

Between 10 and 11 o'clock, on the night of the 19th of June last, I was called to see Mrs. S., aged about forty-seven years, who had been purging and vomiting at intervals of half an hour, or less, during the whole afternoon, and was then suffering much from cramps. I ordered the following:—

℞ Submur. Hydrarg. gr.xxiv,
Pulv. Opii, gr.x,
Plumbi Acetat. gr.viii,
Gum Camphor, gr.xii,
m. ft. pill no. 6. Two to be given every hour.

The following morning I found her much easier, the cramp mitigated, the vomiting and purging less frequent, but great prostration. She had taken but four of the pills during the night. I now directed forty drops of the following, every half hour with brandy.

℞ Tinct. Catechu,

" Krameriæ, à à ʒi,

" Opii,

Sp. Ammoniæ, à à ʒi.

In the evening I found her much revived; the purging and vomiting had ceased though there was considerable nausea, but no cramp. For the next two days she had no discharge from the bowels, complained of but little nausea, her pulse quick, weak and threadlike, and her skin moist and nearly natural. On the evening of the fourth day from the attack she complained that her left hand and wrist had not felt natural since the cramp in it, that the cramp in that hand had been most severe. Upon examination I could detect nothing in the appearance of the hand and arm or in its temperature to justify any course of treatment.

Five days from the commencement she expressed herself well, feeling a little weak, her bowels in good condition and her pulse pretty good. Deeming her convalescent I ceased to call.

Four days after I was again called to see her. She was suffering from great pain in the right hypochondrium, some fullness of the stomach and tenderness of the bowels, pulse full and quick, tongue coated with dark brown, and considerable heat of the skin. The discharges from the bowels were of a gray-ashy color. I di rected calomel and opium to be given, and a blister to be applied over the pain, to be followed by a poultice. In the evening her pulse was rather weak, skin moist; my directions had not been followed, but in part, owing to the family being unable to procure the medicines. She now complained more of her hand and arm than she had formerly done; the wrist feeling stiff. There were at times quick, shooting pains along the course of the vessels from the elbow to the ends of the fingers. On examination I found her hand and arm quite cool, the ends of the fingers particularly. I directed her arm and hand to be bathed in warm spirits, and the application of a stimulating liniment, with friction. For the next two days I observed but little change in her condition.

July 1st. Her bowels had been moved pretty freely, the discharges more natural; no tenderness of the bowels; complained less of her hand and arm.

2nd. No pain in the side, pulse pretty good; arm and hand cold and moist, with slight pain at intervals.

3rd. Complained of great pain in the arm and hand as far up as the elbow; to touch or move it produced the most excrutiating pain. The ends of her fingers had assumed a dark-blue color and were very cold; there was some swelling of the wrist and palm of the hand. A swelling was to be felt and distinctly observed on the right lobe of the liver, and there was some tenderness of the first dorsal vertebra. In a few days by proper treatment the swelling disappeared and a healthy action of the liver was set up.

During the ten days following the patient though rather weak, appeared well in every respect but her hand and arm. The swelling progressed slowly but steadily until it reached the elbow; the fingers to the second joint were dead, assuming a dark and dry appearance, and were quite hard; the hand and arm were of a dark reddish cast, cold and humid; destruction of the limb appeared inevitable, notwithstanding the whole catalogue of remedies, stimulant, excitant, &c., had been applied. Quinine, opium, iron, gentian, and strychnia, had been given separately and combined, but afforded no relief. No pulsation of the artery could be perceived in the arm either above or below the elbow.

Amputation was now considered the only alternative, and that was thought to afford but little hope of recovery.

On the 17th the parts began to slough and a line of separation between the sound and diseased parts was to be seen obscurely, but sufficiently distinct to warrant amputation, which was accordingly done on the morning of the 18th with the assistance of Drs. BARTLET, HUNDLEY, and OWEN. Ten days have since elapsed and the patient is doing well with every prospect of recovery.

An examination of the amputated limb revealed nothing of the cause of the disease. The arteries were contracted to half their original diameter, and were coated thinly with blood, but upon separating this, no evidence of inflammation was visible. The veins appeared to be in a healthy condition.

July 27th, 1854.

